# Fabrication and characterization of polysaccharide-based microspheres for ciprofloxacin delivery with controlled release and antimicrobial activity

**DOI:** 10.1039/d5ra06745f

**Published:** 2025-10-20

**Authors:** Qurat ul Ain Ameer, Shazia Akram Ghumman, Huma Hameed, Sobia Noreen, Shazia Noureen, Rizwana Kausar, Ali Irfan, Katarzyna Kotwica-Mojzych, Fozia Batool, Mariola Glowacka, Maria Rana, Mariusz Mojzych, Yousef A. Bin Jardan

**Affiliations:** a College of Pharmacy, University of Sargodha Sargodha-40100 Pakistan drquratulainameer@gmail.com shazia.akram@uos.edu.pk; b Faculty of Pharmaceutical Sciences, University of Central Punjab Lahore 54000 Pakistan huma.hameed@ucp.edu.pk; c Institute of Chemistry, University of Sargodha Sargodha 40100 Pakistan sobia.noreen@uos.edu.pk shazianoureen11@gmail.com fozia.batool@uos.edu.pk; d ILM College of Pharmaceutical Sciences Sargodha 40100 Pakistan rizvi_awan@yahoo.com; e Department of Chemistry, Government College University Faisalabad Faisalabad-38000 Pakistan raialiirfan@gmail.com; f Department of Histology, Embryology and Cytophysiology, Medical University of Lublin Radziwillowska 11 20-080 Lublin Poland katarzyna.kotwica-mojzych@umlub.pl; g Faculty of Health Sciences, Collegium Medicum, The Mazovian Academy in Plock Pl. Dabrowskiego 2 09-402 Plock Poland m.glowacka@mazowiecka.edu.pl m.mojzych@mazowiecka.edu.pl; h Riphah Institute of Pharmaceutical Sciences, Riphah International University Lahore Campus Pakistan maria.rana@riphah.edu.pk; i Department of Pharmaceutics, College of Pharmacy, King Saud University Riyadh 11451 Saudi Arabia ybinjardan@ksu.edu.sa

## Abstract

In this study, ciprofloxacin (CIP)-loaded microspheres were formulated using alginate and cress seed mucilage as natural polymeric carriers; microspheres were prepared *via* ionic gelation, facilitating the effective encapsulation of CIP. The swelling kinetics of the microspheres were evaluated under different physiological pH conditions (1.2, 6.8, and 7.4) to simulate the acidic and intestinal environments. The results showed minimal swelling at pH 1.2, while significantly higher swelling was observed at pH 6.8 and 7.4, indicating pH-responsive behavior. Characterization and *in vitro* release studies confirmed the successful incorporation of CIP into the microspheres without compromising their functional properties. The Korsmeyer–Peppas model was the best fit for drug release data, indicating that ciprofloxacin release followed a non-Fickian (anomalous) diffusion pattern, representing a synergistic contribution of both diffusion and polymer chain relaxation mechanisms. The microspheres exhibited promising potential for drug delivery, while antibacterial assays demonstrated a significant inhibitory effect, attributed to the sustained release of ciprofloxacin. This study highlights the potential of alginate and cress seed-based microspheres as efficient carriers for the controlled release of ciprofloxacin.

## Abstract 2

In this study, ciprofloxacin (CIP)-loaded microspheres were formulated using alginate and cress seed mucilage as natural polymeric carriers; microspheres were prepared *via* ionic gelation, facilitating the effective encapsulation of CIP.

## Graphical abstract

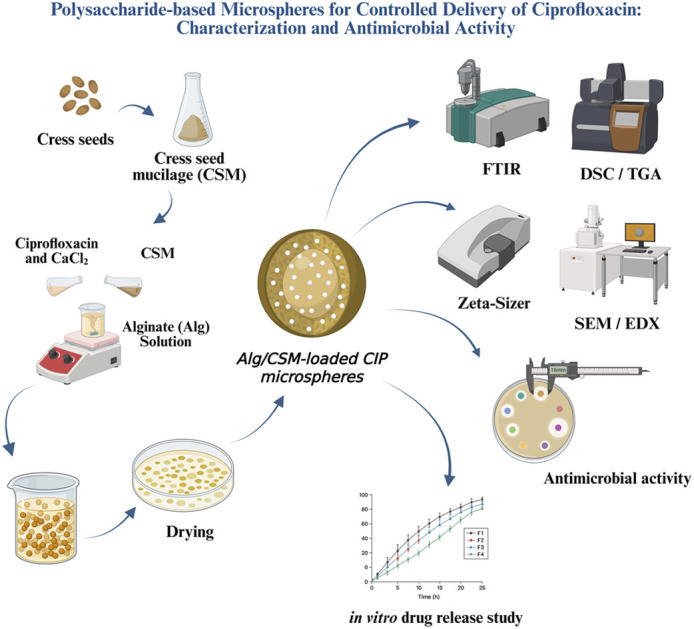

## 1 Introduction

Ciprofloxacin (CIP), a third-generation quinolone antibiotic, exhibits broad-spectrum antibacterial effects and targets various bacterial pathogens by inhibiting DNA replication, ultimately resulting in cell death.^1^ This medication has been shown to inhibit bacterial variants, including *Enterobacter* and *Pseudomonas aeruginosa*, and manage various types of infections, such as those affecting the urinary tract, gonorrhea, bones, skin, and lungs (pneumonia).^2^ CIP administration necessitates meticulous dose assessments, cautiously tailored to pursue the precise infectious disease being treated and to achieve premier healing outcomes.^3^ The suggested dose for efficient healing of intestinal infections is 1 g, administered twice a day for 7 days. For additional intense infections, together with bone and joint infections, a better dose of 1.0–1.5 g is given 2–3 times in step with day for 4–6 weeks or longer.^4^ A parallel treatment protocol of 1.0–1.5 g twice daily for 1–2 weeks is applied for skin and soft tissue infections and pneumonia. CIP has a short half-life of just 3–5 hours,^5^ so repetitive delivery of CIP may additionally result in various detrimental outcomes, consisting of nausea, vomiting, diarrhea, indigestion, stomach ache, and anorexia.^6^ Additionally, more severe harmful reactions, encompassing phototoxicity, skin rashes, pruritus, and conjunctival congestion, may also be noted.^7^ Addressing the challenges of ciprofloxacin therapy requires a delivery system capable of providing consistent, prolonged, and controlled drug release. As for this, crafted ciprofloxacin carrageenan complexes have been engineered that achieved sustained release over 6 hours. Concurrently, ciprofloxacin-loaded oral self-emulsifying drug delivery systems were prepared, managed the drug supply was managed for 5 hours.^8^ Irrespective, considerable complications remain in addressing the issues of CIP administration, demanding extended research and development. This research documents the fabrication of polysaccharide-based microspheres entailing sodium alginate and cress seed mucilage, which not only promote the prolonged release of CIP but also mitigate its unwanted effects due to their uncommon biodegradability, lessen toxicity, and antimicrobial interest.

Microspheres are stable, spherical particles measuring significantly less than 1000 micrometers, typically used for drug delivery, and key features comprise consistent size and form, large surface area, biodegradability, and the capability for extended drug release due to their absorbent structure.^9^ These hallmarks make them exemplary for controlled-release formulations, proposing both stability and biocompatibility. Microspheres boost the consistency of drug absorption and reduce nearby irritation commonly related to single-dose formulations.^10^ Besides that, microspheres shield sensitive drugs like ciprofloxacin from gastrointestinal degradation, boosting stability and bioavailability.^11^ The wide range of recognized methods for preparing drug-loaded microspheres consists of solvent evaporation, phase separation, spray-drying, ionic gelation, and *in situ* polymerization. Out of these, the ionic gelation technique is notably opted for because of its economic feasibility, adaptability, and ease of implementation.^12^

Polysaccharide polymers, like alginates, stand out for their exclusive benefits, along with being budget-friendly, regenerative, and more approachable than other macromolecules typically used to put together microspheres.^13^ An additional potential candidate is cress seed mucilage (CSM), a natural polysaccharide sourced from the seeds of *Lepidium sativum*. CSM is renowned for its extraordinary gel-forming ability, excessive viscosity, and considerable water retention capacity.^14^ These attributes create a remarkable match for Na-Alg, especially in upgrading mechanical properties, hastening the gelation rates, and improving the drug release profile, provoking a more controlled system.^15^

The current research sought to explore challenges associated with CIP, including its brief half-life, solubility that varies with pH, the requirement for regular dosing, and a range of adverse side effects. The integration of Alg and CSM presented an encouraging approach to enhance CIP delivery, extend the shelf life and therapeutic effectiveness of the encapsulated API, and address the shortcomings of earlier drug delivery systems. This microsphere system provided a regulated release profile for the medication, successfully prolonging CIP's half-life and lowering daily doses, thereby enhancing patient compliance with the treatment. Integrating CSM into the sodium alginate formulation significantly improved its swelling characteristics, validating the controlled release of CIP. The research findings showed that the microspheres provided dual protection: shielding the stomach from drug-related side effects and preventing CIP degradation in the acidic gastric environment. Furthermore, the antibacterial performance of the microspheres supports their potential for safe, long-term use in managing bacterial infections with minimal toxicity.

## 2 Methodology

### 2.1. Materials and methods

Cress seeds were sourced from the nearby market of Sargodha. Ciprofloxacin was acquired from Scilife Pharma (Pvt) Ltd. Sodium alginate: *M*_w_ 216, CAS-NO.: 9005-38-3, Sigma-Aldrich, calcium chloride (Merck), ethanol (Sigma-Aldrich), potassium dihydrogen phosphate, sodium hydroxide, and sodium chloride (Sigma-Aldrich). Laboratory-grade solvents were utilized throughout the research. The marketed formulation served as a benchmark for comparison.

### 2.2. Extraction of mucilage from cress seeds (CSM)

One hundred grams of cress seeds were thoroughly washed and soaked in 500 mL of distilled water for 12 hours to extract the mucilage. During this time, the seeds became fully hydrated, swelling and becoming coated with a thick layer of mucilage. The entire mixture of swollen seeds and mucilage was blended and then pressed through muslin fabric to separate the aqueous mucilaginous extract. The polysaccharides in the mucilage were precipitated using 96% ethanol. The mucilage and ethanol were gradually mixed in a 1 : 3 ratio (mucilage to ethanol), resulting in precipitation. The precipitated mucilage was collected and dried on glass plates in a hot air oven at 38 °C until completely dry.^16^ Isolated mucilage was evaluated for physicochemical properties and chemical characterization. The physicochemical properties include pH, solubility, viscosity, particle size, and loss on drying. The chemical characterization of cress seed mucilage involved various chemical tests, including Molisch's test, ruthenium test, and iodine test, to confirm the presence of mucilage in the extracted material.^17,18^

### 2.3. Fabrication of Alg/CSM-loaded CIP microspheres

Microspheres were formulated using the ionic gelation technique. Sodium alginate (Alg) was dispersed in distilled water with stirring maintained at 300 rpm, followed by the addition of cress seed mucilage (CSM) to achieve a uniform polymer blend. CIP was dissolved in 0.1 N HCL solution to ensure complete solubility of the drug before adding it to the polymer mixture. The drug–polymer mixture was slowly added dropwise through a 24-gauge syringe to the 100 mL of 7% w/v calcium chloride solution, to allow the formation of microspheres. Then, the microspheres were separated and spread over filter paper to dry for 24 hours in air. Following drying, the microspheres were kept in a sealed amber glass bottle.^19^ The formulations were coded as F1–F4 based on varying polymer ratios of alginate and cress seed mucilage (CSM) while keeping the drug concentration constant, as presented in Table 3. The purpose was to evaluate the effect of polymer composition on the encapsulation efficiency, particle size, swelling behavior, and drug release profile of the microspheres.

### 2.4. Evaluation of Alg/CSM-loaded CIP microspheres

#### 2.4.1. Fourier transform infrared spectroscopy (FTIR) analysis

The microsphere sample was crushed into a fine powder and placed into the DRIFTS sample holder. FTIR spectroscopic analysis was conducted using an IR Prestige-21 Shimadzu, Germany. The spectra were acquired across the wavelength range of approximately 4000–400^−1^.^20^

#### 2.4.2. DSC and TGA analysis

DSC/TGA investigation was conducted to assess the stability of the polymer, drug, and formulated microspheres. Accurately weighed 5–10 mg of sample and tightly sealed aluminum pans, and a flow rate of 10 °C over the range of 20 °C to 700 °C, maintained an inert environment by purging N_2_ gas at a rate of 20 mL min^−1^.^21^

#### 2.4.3. XRD analysis

X-ray diffraction (XRD) analysis was performed using a Rigaku MiniFlex X-ray Diffractometer with Cu Kα radiation (*λ* = 1.5418 Å). The samples, finely ground into powder, were placed on a zero-background silicon sample holder. The XRD patterns, presented as diffractograms with 2*θ* angles on the *x*-axis and diffracted intensity on the *y*-axis, provided a comprehensive view of the crystalline structures.^22^

#### 2.4.4. SEM-EDX analysis

The surface morphology of Alg/CSM-loaded CIP microspheres was examined using scanning electron microscopy (SEM), using JEOL JSM IT100, and elemental composition was determined using energy-dispersive X-ray (EDX).^23^

#### 2.4.5. Particle size analysis

An optical microscope (Olympus) was used to determine the size of dried Alg/CSM-loaded CIP microspheres. A small quantity of microspheres (a few milligrams) was placed on the ocular micrometer that was formerly calibrated with the stage micrometer.^24^

#### 2.4.6. Drug encapsulation efficiency, drug loading, and percent yield

100 mg of Alg/CSM-loaded microspheres were collected, ground into fine powder, put into a 250 mL volumetric flask, and phosphate buffer of pH 6.8 was added up to 250 mL. The mixture was set aside for 24 hours at 37 ± 1 °C with periodic stirring. After 24 hours, the mixture was stirred for 15–20 min at 500 rpm. The polymer was filtered using a Whatman filter, and the filtrate was examined by UV spectrophotometry under a detection wavelength of 274 nm. The encapsulation efficiency (%) of microspheres (*n* = 3) was calculated by using the following formula.
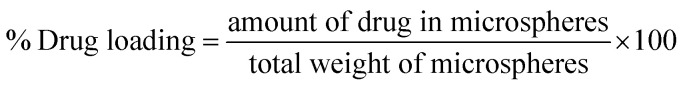

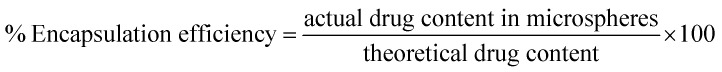


To access the percentage yield of the Alg/CSM-loaded CIP microspheres, the practical yield was compared with the theoretical yield and expressed as a percentage. While the practical yield refers to the end weight of the dried microspheres, the theoretical yield accounts for the full weight of the input materials, including both the drug and the polymer.^25^ The percentage yield was calculated using the following formula;
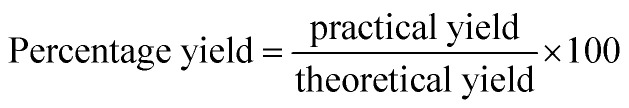


#### 2.4.7. Swelling ability

The swelling behavior of the Alg/CSM-loaded CIP microspheres was evaluated by immersing them in 100 mL of media at pH 7.4, 6.8, and pH 1.2, using a USP dissolution apparatus, operated at a temperature of 37 ± 1 °C and a rotation speed of 50 rpm.^19^ At predetermined time points, the microspheres were withdrawn, gently blotted with tissue paper to remove excess surface liquid, and then weighed to determine the extent of swelling. The swelling index of the microspheres was evaluated using the following formula.
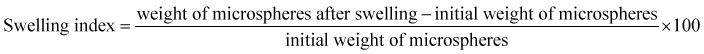


#### 2.4.8. Anti-microbial activity test

Antimicrobial testing was carried out through the disk diffusion technique by placing the sample on the disk, which was placed on an agar plate inoculated with microbial isolates like *Staphylococcus aureus* (ATCC#25923), *Listeria monocytogenes* (ATCC#13932), *Escherichia coli* (ATCC#25922), and *Pseudomonas aeruginosa* (ATCC#10145). The antimicrobial sample diffuses outward, creating a zone where bacteria cannot grow, called the inhibition zone. The size of this zone indicates the effectiveness of the sample against the bacteria.^19,26^

#### 2.4.9. *In vitro* drug release profile

Dissolution testing was conducted to evaluate the release profile of Alg/CSM-loaded CIP microspheres using a USP dissolution apparatus, maintained at 37 ± 1 °C with stirring at 50 rpm. A total of 100 mg of the microsphere was exposed to 900 mL of gastric fluid (SGF) (pH 1.2) for 2 h and then in phosphate buffer (pH 6.8) for 22 h. At predetermined time intervals, samples were withdrawn, and an equal volume of dissolution medium was replenished. Using UV spectrophotometry at *λ*_max_ 274 nm, the drug content in each sample was quantified. Time-dependent cumulative release curves were generated to assess the release kinetics.^27,28^

#### 2.4.10. Drug release kinetic analysis

The release profile data were applied to various mathematical models: zero-order kinetics, first-order kinetics, Higuchi model, Hixson–Crowell, and Korsmeyer–Peppas model, to elucidate the drug release profile.^29^ Different models applied to the drug release are:Zero-order kinetics:*Q*_*t*_ = *Q*_0_ + *K*_0_*t*First-order kinetics:d*C*/d*t* = −*K*_1_*C*Higuchi model:*Q*_*t*_ = *kt*^0.5^Hixson–Crowell model:(*W*_0_^1/3^ − *W*_*t*_^1/3^) = *k*_h_*t*Korsmeyer–Peppas model:*Q*_*t*_/*Q*_∞_ = *k*_p_*t*^*n*^

The value of the release exponent (*n*) is crucial in determining the mechanism of drug release. The values of “*n*” are geometry-dependent and help differentiate among Fickian diffusion, anomalous (non-Fickian) transport, Case II transport, and Super Case II transport.

### 2.5. Statistical analysis

All the collected results were expressed as the mean ± standard deviation after analysis by one-way ANOVA. To evaluate the goodness of fit and predictive accuracy of various kinetic models, statistical parameters such as the coefficient of determination (*R*^2^) and Model Selection Criterion (MSC) were calculated.

## 3 Results

### 3.1. Characterization of isolated mucilage

The yield of isolated mucilage was 26.43% w/w. The mucilage was brownish, odorless, with a smooth and regular texture, and a characteristic taste.^18^ The physicochemical properties are shown in Table 1. The chemical characterization of mucilage was performed using Molisch's test, ruthenium test, and iodine test, and the results are presented in Table 2.

**Table 1:** Physicochemical properties of isolated mucilage

Parameter	Observation
Carr's index	11.2 ± 0.37
Hausner's ratio	1.15 ± 0.24
Angle of repose	22.7 ± 0.15
pH of mucilage	7.2 ± 0.04
Loss on drying	5.2%
Viscosity	206.88 cps
Mean particle size	82.57 ± 0.45 μm
Solubility	Swells in cold water, dissolves in hot water, insoluble in organic solvents

**Table 2:** Chemical characterization of isolated mucilage

Test	Observed	Results
Molisch's test	Purple to violet color	Carbohydrate present
Ruthenium test	Pink color	Mucilage present
Iodine test	Blue/purple color	Polysaccharides present

### 3.2. Fourier transform infrared spectroscopy (FTIR) analysis

**Fig. 1:**
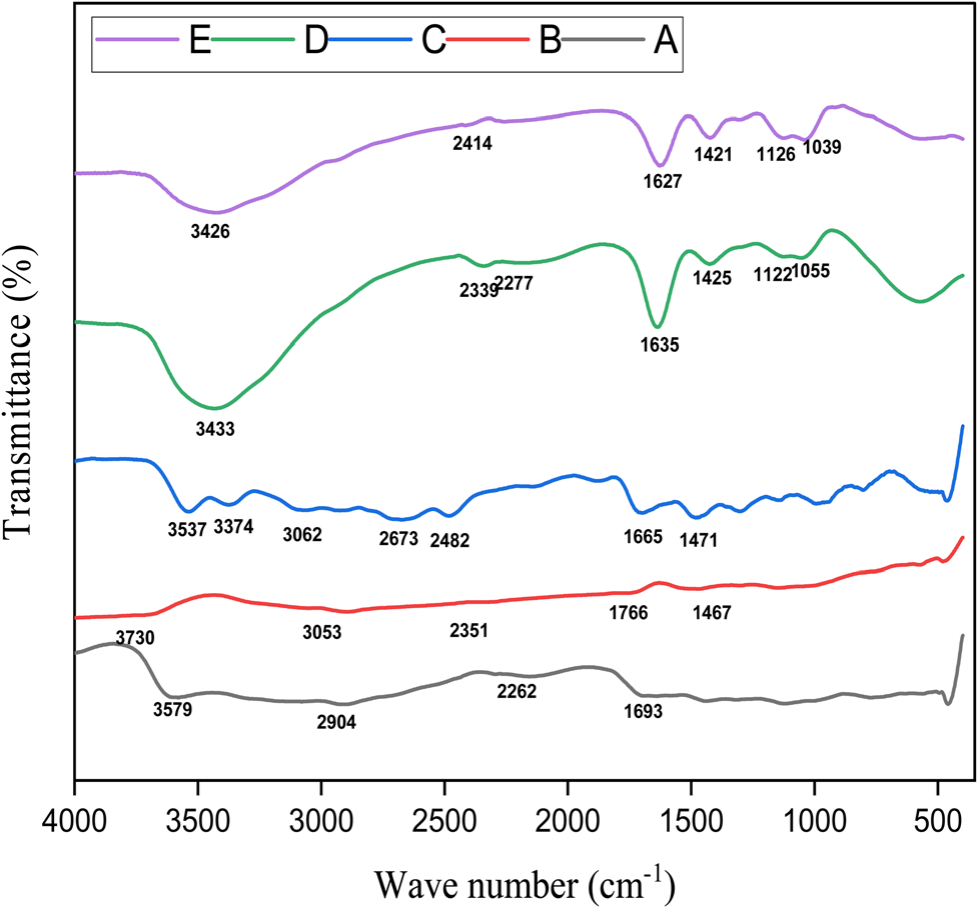
FTIR spectra of (A) CSM; (B) Alg; (C) CIP; (D) Alg/CSM unloaded microspheres; (E) Alg/CSM-loaded CIP microspheres.

The FTIR spectra of CSM, CIP, Alg/CSM unloaded microspheres, and Alg/CSM-loaded CIP microspheres displayed distinct peaks corresponding to their functional groups, as shown in Fig. 1. The FTIR spectrum of CSM displayed (C–O) stretching at 1120 cm^−1^ and (C=O) stretching at 1639 cm^−1^. Peaks in the 1200–1000 cm^−1^ region are characteristic of C–O and C–O–C vibrations, confirming mucilage composition. Furthermore, peaks at 2154 cm^−1^ and 2262 cm^−1^ suggested distinct functional groups or molecular interactions, highlighting the structural complexity of CSM. The broad absorption band at 3200–3400 cm^−1^ confirmed O–H stretching, indicating the presence of polysaccharides.^30^

CIP exhibited its characteristic peaks at 1620 cm^−1^ and 1695 cm^−1^ (C=O), 1139 cm^−1^ and 1149 cm^−1^ (C–O), and 2924 cm^−1^ (C–H), with a broad (O–H) stretching observed between 3537 cm^−1^ and 3851 cm^−1^, indicating strong hydrogen bonding. Additional peaks at 2140 cm^−1^ and 2175 cm^−1^ suggest possible molecular interactions.^31^ The broad peak (∼3500–3300 cm^−1^) represented O–H and N–H stretching (due to carboxyl and amine groups). Sharp peak (∼1700 cm^−1^) of C=O stretching (quinolone ketone group). Bands at ∼1620 and 1450 cm^−1^ of C=C and N–H bending (aromatic ring and amide functionalities). Peaks around ∼1200–1000 cm^−1^ of C–F stretching, characteristic of fluoroquinolone drugs.

The Alg/CSM unloaded microspheres exhibited C–O stretching at 1296 cm^−1^ and C=O stretching at 1469 cm^−1^, a broad O–H stretching region at 3689–3996 cm^−1^, peaks at 2868 cm^−1^ (C–H stretching) and 1861 cm^−1^ (C–H bending), along with peaks at 2187 cm^−1^ and 2279 cm^−1^. The FTIR spectrum of loaded Alg/CSM-loaded CIP microspheres exhibited C–O stretching at 1300 cm^−1^ and C=O stretching at 1450 cm^−1^, along with a broad O–H stretching region between 3601–3952 cm^−1^. Shifts in C–H stretching (3099 cm^−1^) and bending (1674 cm^−1^) were also observed. Additionally, new peaks at 2173 cm^−1^ and 2389 cm^−1^, which are absent in unloaded Alg/CSM microspheres, suggested possible cross-linking interactions. The C=O shift from 1469 cm^−1^ to 1450 cm^−1^, and the broadening of O–H stretching from 3689–3996 cm^−1^ to 3601–3952 cm^−1^ indicated covalent and hydrogen bonding interactions between ciprofloxacin and the polymer matrix.^32^ The reduced intensity of ciprofloxacin peaks in drug-loaded Alg/CSM microspheres confirmed its encapsulation, likely improving controlled release properties.

### 3.3. Thermal analysis: TGA and DSC evaluation

**Fig. 2:**
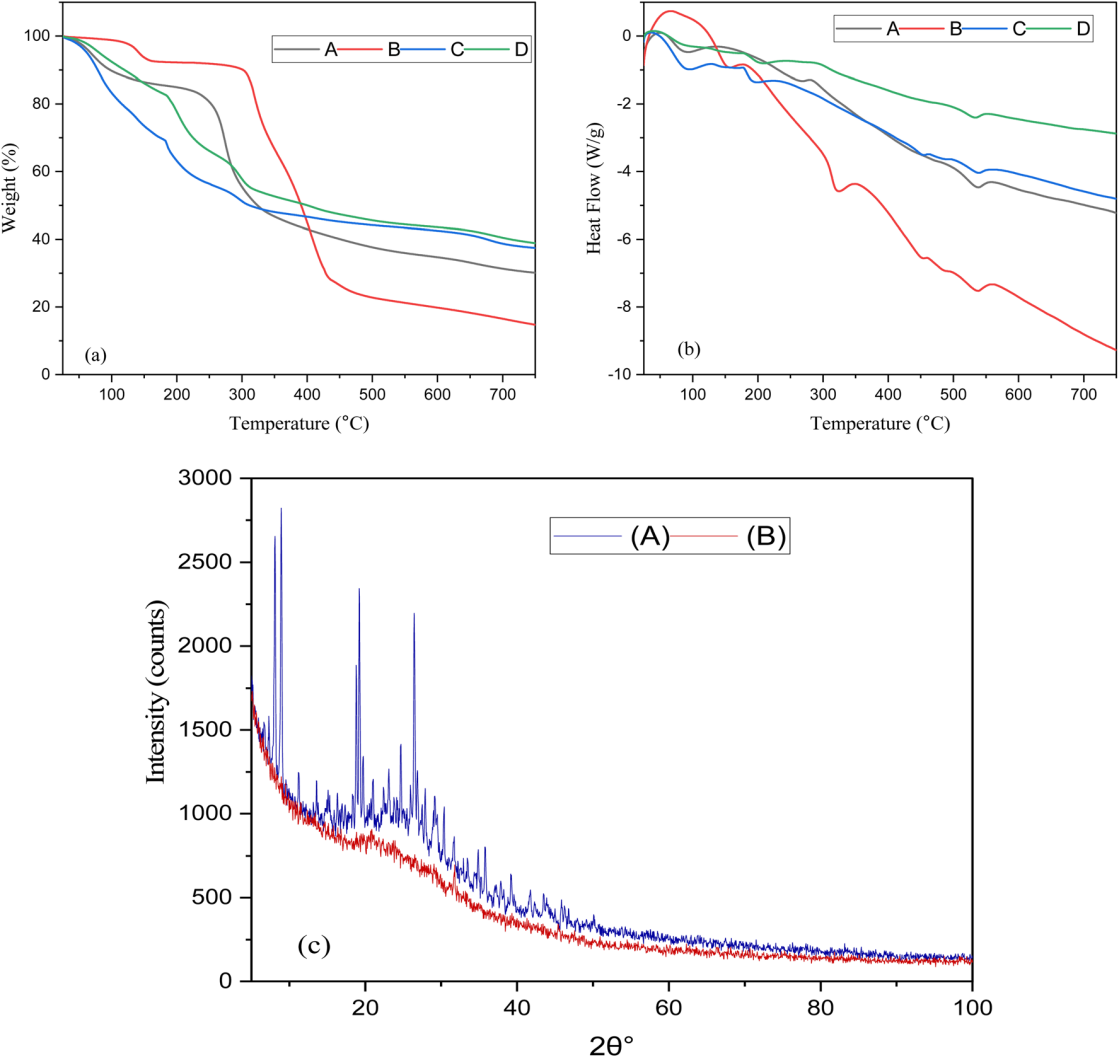
(a and b) TGA and DSC of (A) CSM; (B) CIP; (C) Alg/CSM unloaded microspheres; (D) Alg/CSM-loaded CIP microspheres; (c) XRD of (A) CIP, (B) Alg/CSM-loaded CIP microspheres.

Thermal stability of the individual components and formulations was evaluated using thermogravimetric analysis (TGA), as depicted in Fig. 2a. All samples exhibited multistep degradation patterns, indicating the complex thermal behavior of the biomaterials and drug-loaded systems. CSM (A) showed initial weight loss below 120 °C due to moisture evaporation, followed by significant degradation between 200–350 °C, indicating decomposition of polysaccharides, with a final residue of about 15%, with a peak degradation around 290 °C.^33^ CIP (B) demonstrated comparatively greater stability, exhibiting significant weight loss in the range of 320–450 °C, with a peak degradation temperature near 390 °C.^34^ The unloaded microspheres (C) showed a two-step degradation behavior, with the initial loss occurring below 120 °C from moisture release. The primary degradation phase occurred between 220 °C and 450 °C, peaking around 310 °C, suggesting enhanced thermal stability relative to the mucilage by itself. This improvement showed the formation of a cross-linked polymer network, which offers a degree of thermal resistance.^35,36^ Significantly, loaded microspheres (Sample D) exhibited the greatest thermal stability compared to all other samples. The primary onset of degradation was postponed to nearly 250 °C, with significant weight loss occurring from 300 °C to 500 °C, and the highest degradation temperature moved to roughly 370 °C. This alteration validated the effective encapsulation of ciprofloxacin within the polymer matrix and indicates that the polymeric framework offers a protective impact against thermal degradation.^37^ In general, the comparative change in both the onset and peak degradation temperatures of the loaded microspheres compared to the pure drug and polymer components suggests that the formulation greatly improves the thermal stability of ciprofloxacin. The heightened stability could be due to hydrogen bonding and electrostatic interactions between ciprofloxacin and the biopolymers, which probably prevent early degradation.

Differential scanning calorimetry (DSC) was performed to examine the thermal transitions and interactions between the formulation components. The DSC thermograms (Fig. 2b) showed clear thermal characteristics for the pure components and the microsphere formulations. The CEM (Sample A) showed a wide endothermic peak in the range of 90–110 °C, due to moisture evaporation, and then a slow thermal change between 200–300 °C, reflecting polysaccharide breakdown. The lack of distinct melting points indicated its amorphous characteristics.^38^ The pure CIP (Sample B) showed a distinct and sharp endothermic peak around 320–330 °C, which aligned with its melting point and thermal breakdown. This distinct peak was typical of its crystalline arrangement.^39^ In comparison, the unloaded microspheres (Sample C) exhibited a wide endothermic shift occurring between 80–120 °C, attributed to the evaporation of moisture. Another significant event occurred between 250–350 °C, indicating the breakdown of the cross-linked polymer structure. Importantly, no clear peak was detected for ciprofloxacin, validating the drug's absence and demonstrating effective microsphere creation from natural biopolymers. The Alg/CSM-loaded CIP microspheres (Sample D) showed a broader and displaced endothermic event from 280–370 °C, with a diminished or missing ciprofloxacin melting peak, suggesting that the drug is molecularly dispersed or encapsulated in an amorphous state within the polymeric matrix. This change in thermal events indicated robust interactions, like hydrogen bonding and electrostatic forces, between CIP and the polymer matrix (CSM and Alg). Moreover, the lack of a distinct melting endotherm around ∼320 °C in the loaded microspheres indicated that ciprofloxacin loses its crystalline structure upon encapsulation.^40^ The thermal shielding effect, along with the higher decomposition range of the composite, demonstrated enhanced thermal stability of the drug when integrated into the microspheres.

### 3.4. Powder X-ray diffraction (PXRD) analysis

The crystallinity of the CIP and Alg/CSM-loaded CIP microspheres was evaluated using XRD, as shown in Fig. 2c. This intense peak, 8.95°signified the presence of a highly ordered lattice structure in native CIP. A medium-intensity reflection at 12.40° further confirmed crystallinity. Additional distinct peaks at 15.80° and 20.20°represented long-range molecular order. Sharp, high-intensity peaks at 26.60° and 32.40° further supported the crystalline state of the drug.^41^ These peaks, collectively, confirmed that unprocessed CIP exists in a crystalline form with a well-organized molecular arrangement. Upon encapsulation into the Alg/CSM matrix, the XRD pattern of CIP-loaded microspheres showed a significant alteration: The characteristic CIP peaks at 8.95°, 12.40°, and 15.80° were either greatly reduced in intensity or completely disappeared, indicating disruption of the original crystal lattice. A broad peak at 14.72° emerged, which could indicate partial retention of structure or a contribution from the polymeric matrix. The 20.20° peak was replaced by a broader, lower-intensity reflection at 19.26°, suggesting amorphous dispersion. The 26.60° peak of CIP persisted weakly as 26.48°, but with significantly diminished intensity, implying a partial loss of order.^40^ New peaks observed at 22.82°, 29.88°, 33.14°, and 37.62° are likely attributed to the Alg/CSM matrix, or weak crystalline regions formed during spray drying or gelation, but these were of low intensity and broad nature, characteristic of amorphous or semi-crystalline materials. This peak-by-peak comparison demonstrated that encapsulation caused a significant disruption in the crystallinity of CIP, either by molecular dispersion within the polymeric network or by conversion into an amorphous phase.

### 3.5. Particle size analysis

The particle size analysis revealed notable differences across the formulations, with sizes ranging from 546 ± 0.18 μm to 772 ± 0.43 μm as presented in Table 3. The smallest particles were observed in formulation F4, which had the highest alginate concentration. This may be attributed to the faster gelation rate and stronger ionic crosslinking of alginate with calcium chloride, resulting in tighter, more compact microspheres. Conversely, formulation F2 showed the greatest particle size, likely because of the elevated amount of cress seed mucilage, which often absorbs water and creates larger, looser structures. Intermediate sizes were observed in F1 and F3, indicating that the particle size can be precisely modified by altering the polymer ratios.^29^

**Table 3:** Formulation composition of Alg/CSM microspheres loaded with CIP^a^

Formulations	bCIP (g)	cAlg (g)	dCSM (g)	Yield (w/v%)	eDEE (%) size (μm)
F1	150	0.2	0.4	82.32 ± 0.32	75.89 ± 0.55 658 ± 0.26
F2	150	0.2	0.5	78.87 ± 0.16	71.45 ± 0.67 772 ± 0.43
F3	150	0.4	0.2	87.21 ± 0.28	78.02 ± 0.19 581 ± 0.31
F4	150	0.5	0.2	93.96 ± 0.21	82.81 ± 0.37 546 ± 0.18

a All data are expressed as mean ± S.D.; *n* = 3.

b CIP: ciprofloxacin.

c Alg: alginate.

d CSM: cress seed mucilage.

e DEE: drug entrapment efficiency.

### 3.6. Assessment of drug encapsulation efficiency

The encapsulation efficiency of Alg/CSM-loaded CIP was significantly influenced by the polymer composition in each formulation. DEE values ranged from 71.45 ± 0.67% (F2) to 82.81 ± 0.37% (F4), as shown in Table 3. Formulation F4 exhibited the highest encapsulation efficiency, probably because of the more compact and cohesive gel matrix created, which can efficiently entrap the drug molecules. Conversely, an increased level of cress seed mucilage, observed in F2, could lead to a network that is less cross-linked and more porous, facilitating some drug leakage during the production process. Moderate encapsulation efficiencies were noted in F1 and F3, which had equal or balanced ratios of Alg and CSM. These results underscore the significance of polymeric synergy for maximizing drug retention in the microsphere matrix.^19^

### 3.7. Percent yield

The percent yield of Alg/CSM encapsulated CIP microspheres differed notably across the formulations, influenced by the polymeric ratio of alginate (Alg) and cress seed mucilage (CSM). The yield varied between 78.87 ± 0.16% and 93.96 ± 0.21% as presented in Table 3. The greatest yield was seen in formulation F4, due to the enhanced gel-forming capability and crosslinking effectiveness of alginate when calcium ions are present, resulting in stronger microsphere creation and reduced material loss. These findings suggested that a balanced polymer ratio is crucial for maximizing production output during microsphere formulation.

### 3.8. SEM-EDX analysis

**Fig. 3:**
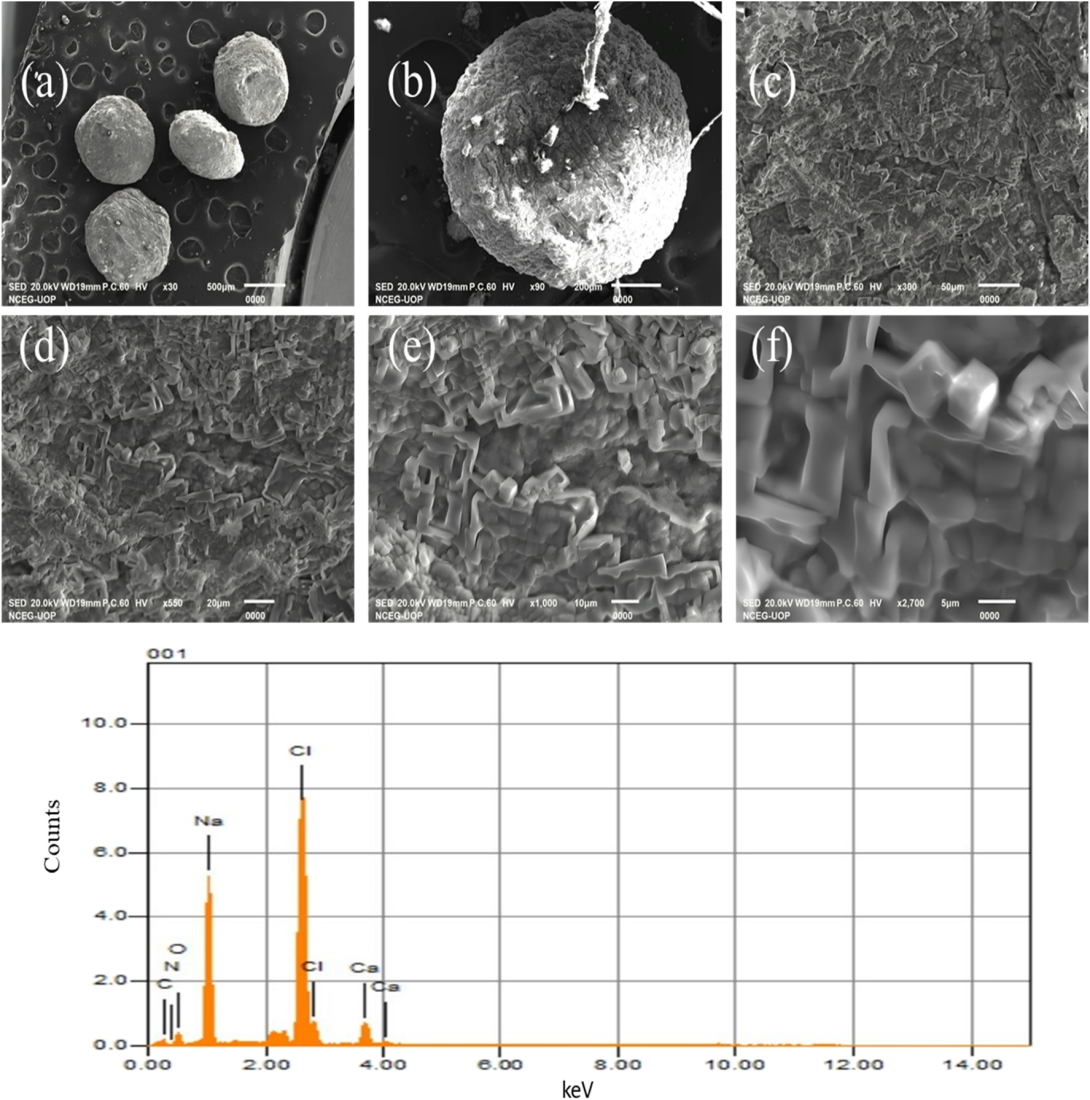
(A) SEM images of Alg/CSM-loaded CIP microspheres (a) 500 μm, (b) 200 μm, (c) 50 μm, (d) 20 μm, (e) 10 μm, (f) 5 μm, and EDX spectrum (B) showing the elemental composition of the ciprofloxacin microspheres.

The SEM images of the Alg/CSM-loaded CIP microspheres (F4), as shown in Fig. 3A, exhibited a distinct morphological profile. The microspheres appeared relatively spherical with a rough, wrinkled, and porous surface, indicative of effective drug encapsulation. The textured outer layer and presence of surface irregularities suggest successful cross-linking, particularly characteristics of alginate-based microspheres. Notably, the observed cracks and deep wrinkles may result from shrinkage during the drying process or solvent evaporation. Images (a) and (b) depict well-formed spherical beads, likely a consequence of the gelling action of Alg and the viscous nature of CSM. The spherical morphology was favorable for controlled drug release, promoting uniform diffusion.^28^ Images (c), (d), and (e) highlight a highly porous and rough surface topology, typical of polymeric drug delivery systems. Such porosity enhances drug entrapment efficiency and modulates the diffusion profile of CIP, supporting a sustained release mechanism. Image (f), captured at higher magnification, revealed the heterogeneous nature of the formulation matrix, potentially reflecting the presence of drug-loaded polymer networks in either crystalline or amorphous form.

The EDX analysis, as presented in Fig. 3B, provided prominent peaks that were observed at approximately 0.27 keV for carbon (C), 0.39 keV for nitrogen (N), and 0.52 keV for oxygen (O)-elements primarily originating from the organic constituents of the matrix, including polysaccharides and ciprofloxacin. A strong signal at 1.04 keV corresponds to sodium (Na), confirming the presence of sodium alginate. The chlorine (Cl) peak appeared around 2.62 keV, serving as a signature of ciprofloxacin. Additionally, a minor peak at 3.69 keV indicated the presence of calcium (Ca), likely arising from trace impurities or residual cross-linking agents.^42^ The detailed elemental composition of the Alg/CSM-loaded CIP microspheres revealed the presence of key elements, confirmed the successful incorporation of both the drug and polymeric carriers. Carbon (C) was detected at 31.54 at%, attributed to the organic nature of the formulation components, including cress seed mucilage, sodium alginate, and ciprofloxacin. Nitrogen (N), present at 2.65 at%, indicates the incorporation of ciprofloxacin, as nitrogen is a characteristic element in its molecular structure. Oxygen (O) content was 15.59 at%, consistent with the polysaccharide backbones of alginate and mucilage, as well as functional groups within ciprofloxacin. Sodium (Na), observed at 25.03 at%, confirmed the presence of sodium alginate as a primary polymeric matrix component. Chlorine (Cl), present at 22.53 at%, is a distinctive marker for ciprofloxacin, further supporting its successful entrapment within the microspheres.^43^ Calcium (Ca), detected at 2.66 at%, may be attributed to residual cross-linking agents or inherent impurities within the alginate. Overall, the EDX results validated the presence of CIP, Alg, and CSM within the microsphere matrix. The elemental profile aligns with the expected composition, supporting the successful formulation of a polymer-based drug delivery system.

### 3.9. pH responsive swelling behavior

**Fig. 4:**
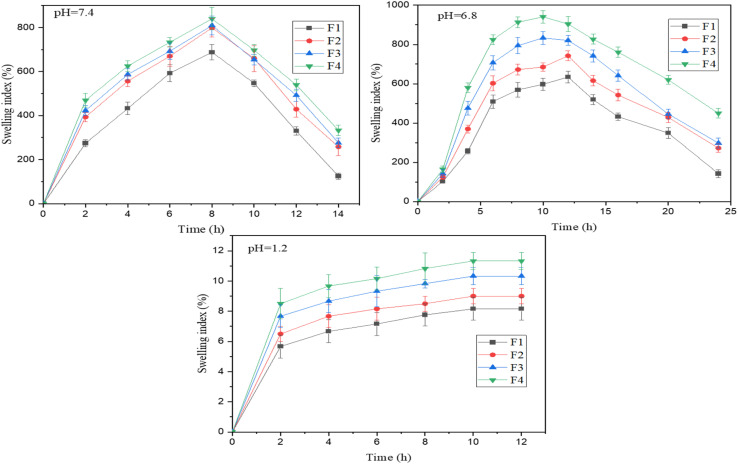
Swelling kinetics of microspheres (F1, F2, F3, and F4) at pH 1.2, pH 6.8, and pH 7.4. Values are represented as mean ± S.D. (*n* = 3).

The swelling behavior of the four formulations (F1–F4) was evaluated at 0.1 M HCl (acidic buffer-pH 1.2), at PB (basic buffer, 6.8 and 7.4), as shown in Fig. 4. As the microspheres contained Alg and CSM, Alg is a polyanionic polymer made of guluronic (G) and mannuronic (M) acids with carboxyl (–COO^−^) groups. At acidic pH, the carboxyl group protonates to –COOH, reducing the negative charge, which leads to poor swelling of microspheres. CSM is also a polysaccharide with hydroxyl and carboxyl groups; chain relaxation and hydration were minimized, and poor swelling was observed at pH 1.2. At a basic pH of 6.8, formulated microspheres exhibited significant swelling; carboxylic acid groups of both Alg and CSM deprotonated to (–COO^−^), leading to electrostatic repulsion between polymer chains, which led to water penetration and polymer relaxation, resulting in higher swelling. CSM is also highly hydrophilic due to its hydroxyl and uronic acid groups, enabling water absorption.^44^ Together, they form a hydrogel matrix with balanced flexibility and water uptake capacity, with high swelling at 12 hours. Their combined hydrophilic nature results in a marked increase in swelling index up to 800–900%, which was maintained for several hours before gradually declining due to structural relaxation and potential matrix breakdown. This behavior highlights their potential for controlled drug release in intestinal environments where such pH levels are physiologically relevant. At a pH of 7.4, the carboxylic groups (–COOH) of alginate and CSM are fully ionized to (–COO^−^), with high swelling indices at 8 hours. The combination of CSM's high hydrophilicity and alginate's structural integrity leads to consistent and robust swelling, followed by a gradual decrease due to matrix erosion^45^. This behavior suggests strong potential for colon-targeted or sustained-release drug delivery systems operating in intestinal pH conditions.

### 3.10. *In vitro* release profile

**Fig. 5:**
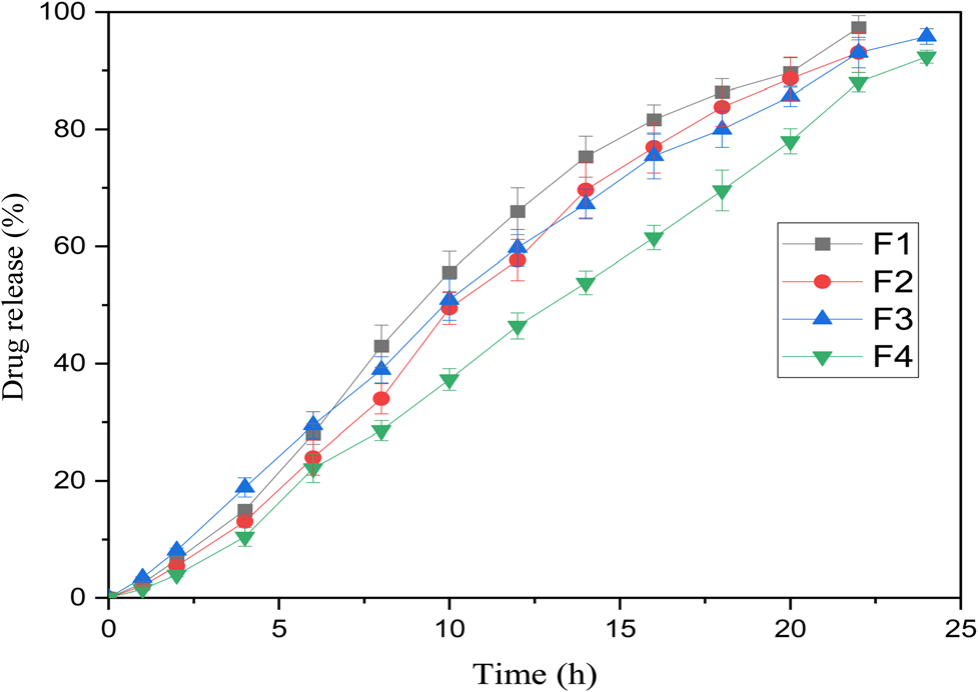
*In vitro* drug release profile of microspheres (F1, F2, F3, and F4) at various pH conditions (1–2 h at pH 1.2 and 22 h at pH 6.8). Values are represented as mean ± S.D. (*n* = 3).

The *in vitro* drug release profiles of all formulations (F1–F4) were evaluated at pH 6.8 over 24 hours to simulate intestinal conditions, and the results are presented in Fig. 5. All formulations exhibited a sustained release behavior, characterized by an initial lag phase followed by a gradual and progressive increase in drug release over time.^46^ Among the formulations, F4 demonstrated the most sustained cumulative drug release, achieving approximately 92% release at 24 hours, indicating that F4 has a more dense matrix system, though F3 exhibited slightly moderate release of approximately 95% at 24 hours. In contrast, F1 and F2 exhibited comparatively fast release rates, particularly during the initial 06–14 hours, indicating higher porosity, showing faster drug diffusion. Notably, F4 showed a slightly lower release profile, highlighting the synergistic role of polymer blend optimization in modulating the release.

### 3.11. Drug release kinetics

The *R*^2^ and MSC values of the models were determined using DDSolver software, and the results are shown in Table 4. The release kinetics indicated that the formulated microspheres followed the Korsmeyer–Peppas model (*R*^2^ = 0.9816–0.9972), a non-Fickian (anomalous) diffusion mechanism, governed by a combination of drug diffusion and matrix relaxation/erosion, as indicated by the progressive and controlled nature of the release curves.^47^ Collectively, these findings highlighted the significant impact of polymer composition, specifically the ratio of Alg to CSM, on the modulation of ciprofloxacin release, offering valuable insights for the rational design of sustained-release drug delivery systems.

**Table 4:** Release kinetic studies of the *in vitro* drug release data of Alg/CSM-loaded CIP microspheres

Model	Parameter	F1	F2	F3	F4
Zero-order model	*R* ^2^	0.9765	0.9871	0.9666	0.9934
	MSC	3.4234	4.0292	3.0763	4.7365
First-order model	*R* ^2^	0.9444	0.9367	0.9533	0.9230
	MSC	2.5606	2.4465	2.7430	2.2830
Higuchi model	*R* ^2^	0.8815	0.8605	0.8958	0.8306
	MSC	1.8041	1.6554	1.9390	1.4939
Korsmeyer–Peppas model	*R* ^2^	0.9816	0.9873	0.9786	0.9972
	MSC	3.5133	3.8959	3.3803	5.4473
	*n*	0.892	0.974	0.840	1.112
Hixon–Crowell model	*R* ^2^	0.9715	0.9649	0.9760	0.9509
	MSC	3.2294	3.0356	3.4064	2.7321

### 3.12. Antibacterial activity of alginate/CSM-loaded microspheres of CIP

**Fig. 6:**
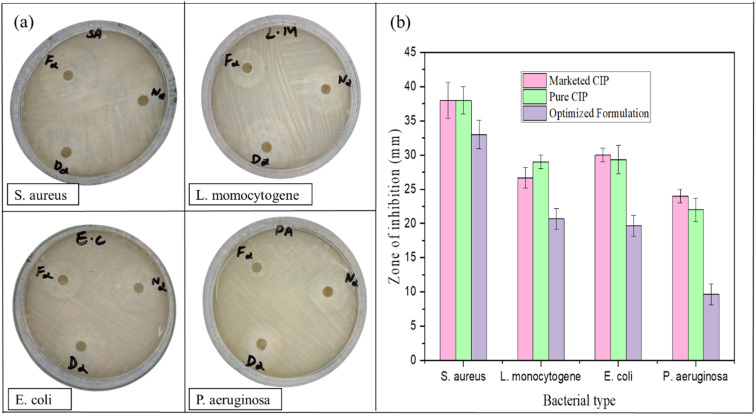
(a) Inhibition zone showing antibacterial activity of different samples against the mentioned strains; N = marketed drug; D = drug (CIP); F = optimized formulation. (b) Quantitative comparison of antibacterial activity showing the zone of inhibition (mm); Values are represented as mean ± S.D. (*n* = 3).

The comparative antimicrobial activity of the three tested formulations, N refers to the marketed drug, F denotes the optimized formulation, and D represents pure drug (CIP), against *Staphylococcus aureus*, *Listeria monocytogenes*, *Escherichia coli*, and *Pseudomonas aeruginosa* revealed significant insights into the influence of formulation type on antibacterial efficacy as shown in Fig. 6.^48,49^ The rationale for including these samples is to provide a comparative assessment of antibacterial efficacy among the optimized formulation, the pure drug, and the marketed reference.

The data clearly show that D, the pure drug CIP, exerted the strongest inhibitory effect across all four strains. This is not unexpected, as ciprofloxacin is a well-established, broad-spectrum fluoroquinolone antibiotic with high water solubility and rapid diffusion properties. The zone of inhibition for D was highest for *E. coli* (31 mm), followed closely by *S. aureus* (40 mm), *L. monocytogenes* (30 mm), and *P. aeruginosa* (23 mm), indicating its potency against both Gram-positive and Gram-negative organisms.^49^ These results reflected the direct availability of the active pharmaceutical ingredient (API) in its most bioactive form, enabling it to reach and act on the bacterial cells without delay or barrier.

The marketed formulation (N) demonstrated antibacterial activity nearly comparable to that of pure CIP. It produced identical zones of inhibition (40 mm) against *S. aureus* and showed only slightly lower activity against *E. coli* (30 mm), *L. monocytogenes* (28 mm), and *P. aeruginosa* (25 mm). The slight reduction in activity compared to D may be attributed to the presence of formulation excipients, pH modifiers, or stabilizers in the commercial product, which may influence drug release or diffusion during the agar diffusion assay. Nonetheless, the high levels of inhibition confirmed that it maintained the therapeutic potency of CIP and was suitably optimized for immediate release and clinical effectiveness.

In contrast, F, the optimized formulation, exhibited considerably lower antimicrobial activity against all tested strains. The inhibition zones were significantly reduced: 35 mm for *S. aureus*, 22 mm for *L. monocytogenes*, 21 mm for *E. coli*, and just 11 mm for *P. aeruginosa*. This reduced activity is most likely on account of the encapsulation of the drug within a polymeric framework-such as Alg or mucilage, which restricts the immediate release of the drug into the surrounding medium. In diffusion-based assays like the agar well diffusion method, only the drug that is readily available in solution can exert its antibacterial effect. Microspheres are designed for sustained or controlled release, which benefits prolonged drug exposure *in vivo* but limits short-term bioavailability in *in vitro* settings. The lower inhibition zones, therefore, do not necessarily indicate reduced efficacy but rather slower drug release, which can be therapeutically advantageous by maintaining prolonged plasma or tissue concentrations, reducing dosing frequency, and improving patient compliance.

The particularly low zone of inhibition against *P. aeruginosa* (11 mm) in the F group is noteworthy. *P. aeruginosa* is a highly resistant Gram-negative pathogen with a robust outer membrane and multiple drug-efflux systems, making it less susceptible to antibiotics unless they are present in sufficient concentration. Since the microsphere system releases the drug gradually, the initial ciprofloxacin concentration may have been insufficient to breach the defense mechanisms of *P. aeruginosa*, resulting in minimal growth inhibition. Overall, the data demonstrate that while pure ciprofloxacin and its marketed formulation offer rapid and potent antibacterial action, microsphere-based delivery systems offer a different advantage-controlled and sustained release. This proposed that while microspheres may show lower zones of inhibition in short-duration *in vitro* assays, they could be more effective in long-term *in vivo* applications, especially for chronic infections requiring steady drug levels. These findings emphasized the importance of interpreting antimicrobial data in the context of formulation design and intended clinical application.

## 4 Conclusion

This work presents a novel bio-polymeric approach to sustained antibiotic delivery through the development of ciprofloxacin-loaded microspheres using a synergistic blend of alginate and cress seed mucilage. The microspheres demonstrated not only structural integrity and uniform morphology, as evidenced by SEM and EDX, but also thermal and physicochemical compatibility confirmed through DSC, TGA, and FTIR analyses. The particle size distribution remained within the optimal range for controlled delivery applications, supporting prolonged drug residence. The release kinetics of the microspheres demonstrated a pronounced non-Fickian, sustained release profile, accompanied by significant antibacterial activity. Altogether, these findings position the Alg-CSM matrix as a biocompatible, eco-friendly, and efficient carrier for the prolonged delivery of ciprofloxacin.

## Author contributions

Qurat ul Ain: conceptualization, methodology, formal analysis, writing – original draft, data curation, investigation; Shazia Akram Ghumman: conceptualization, supervision, project administration, investigation; Huma Hameed: methodology, validation, visualization, writing – review & editing; Sobia Noreen: validation, resources, software, writing, review & editing; Shazia Noureen: data curation, visualization; review & editing; Rizwana Kausar: formal analysis, writing – review & editing; Ali Irfan; formal analysis, funding acquisition, visualization, writing – review & editing; Katarzyna Kotwica-Mojzych: data curation; funding acquisition, investigation, writing – review & editing; Fozia Batool: software analysis, data curation, visualization; writing, review & editing; Mariola Glowacka; formal analysis, investigation, writing, review & editing; Maria Rana: validation, resources, data curation; Mariusz Mojzych: project administration, funding acquisition, formal analysis, investigation, writing – review & editing; Yousef A. Bin Jardan: funding acquisition, formal analysis, visualization; project administration, writing – review & editing. All authors have read and agreed to the published version of the manuscript.

## Conflicts of interest

The authors have no conflicts of interest to declare.

## Data Availability

All the data of this study are contained in the manuscript. For any additional data or information needed regarding this research, please get in touch with the corresponding author, Shazia Akram Ghumman.
